# New horizons in nanoelectrochemistry: concluding remarks

**DOI:** 10.1039/d4fd00183d

**Published:** 2024-12-12

**Authors:** Andrew G. Ewing

**Affiliations:** a Department of Chemistry and Molecular Biology, University of Gothenburg Gothenburg 41350 Sweden andrewe@chem.gu.se

## Abstract

The aim of this paper is to overview the meeting on New horizons in nanoelectrochemistry held at Nanjing University in China in October 2024 and to give some perspective to the work presented. This paper is based on my summary talk and breaks down the subjects in the following areas of nanoelectrochemistry presented at the meeting: nanowires, nanonets, and nanoarrays; nanopores; nanopipettes; spectroelectrochemistry, scanning ion-conductance microscopy and light-active processes at nanointerfaces; scanning electrochemical microscopy and scanning electrochemical cell microscopy; and nanosensors. I end with some discussion of online meetings and where the field might go including artificial intelligence and by asking AI to define the challenges and future of nanoelectrochemistry.

## Abstract 2

The aim of this paper is to overview the meeting on New horizons in nanoelectrochemistry held at Nanjing University in China in October 2024 and to give some perspective to the work presented.

## Graphical abstract

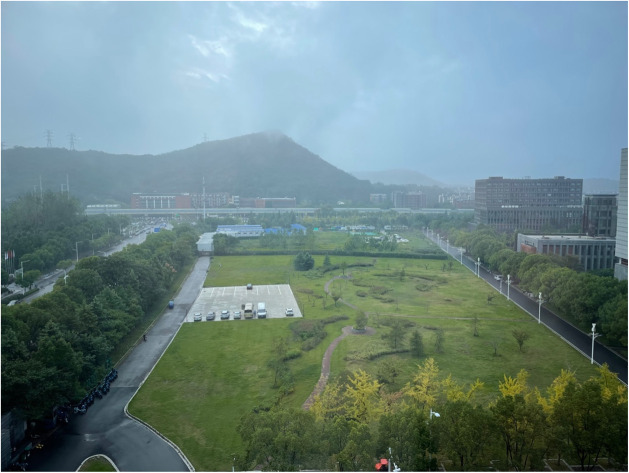

## Introduction

What a great meeting. I will start by thanking the organizers, Yitao Long and Patrick Unwin, for the opportunity to take on this monumental task of summarizing the meeting and putting it into perspective on the fly. I also thank the host helpers at the meeting for making it run. The meeting opened in Nanjing China this year after the last meeting was online owing to the pandemic. Copying the format of Paul Bohn from 2018 (ref. 1), I include a photo of the beautiful view from my hotel room on the Nanjing University campus.
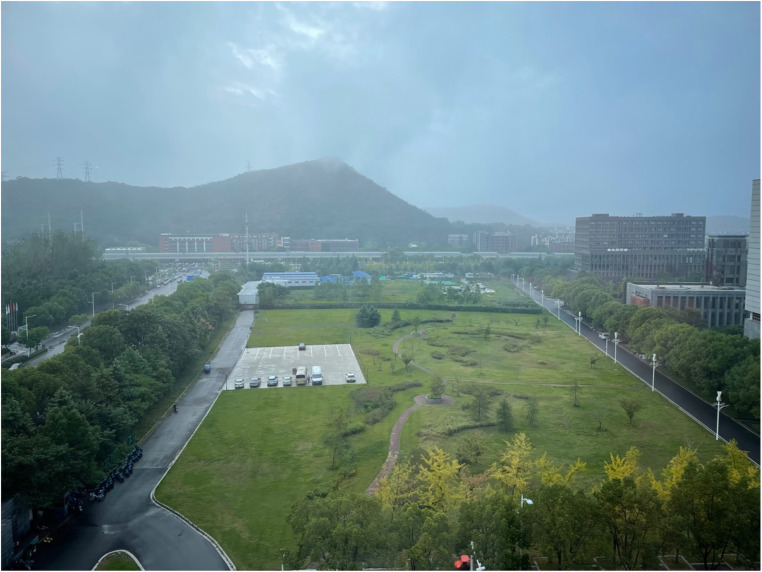


It is difficult to summarize (and critique) a dynamic meeting with a lot of great, dynamic and diverse science like this one, but I will touch on parts of these as I move through this brief summary.

Modern nanoelectrochemistry is becoming an interdisciplinary field at the intersection of nanotechnology and electrochemistry. The history of nanoelectrochemistry up to 2022 was thoroughly reviewed by the previous summary speaker, Patrick Unwin of Warwick University.^2^ In that review, he discussed the historical roots of nanoelectrochemistry, from Volta's work in 1799/1800 on the battery pile, to Wollaston on microelectrodes around the same time, even suggesting he might have made a nanoelectrode. He then characterized the work leading to modern nanoelectrochemistry by periods of time, calling them the Boomer Generation (1946–1964), Generation X (1965–1980), Millenial (1981–1995), Generation Z (1996–2010) and finally, Generation Alpha (2011–2022). Although modern metal and carbon fiber microelectrodes were invented in the late 1970s, they did not really become widely significant until the Millenial age in the 1980s.^3,4^ In 1994, Hoch, Craighead and Jelinski organized what was one of the early microfabrication meetings in Hawaii, “A Symposium on Nanofabrication and Biosystems: Frontiers and Challenges.” To this author this was a precursor to much of the nanofabrication and nanoelectrochemistry revolution. A program for this meeting can be found in the US Archives.^5^ The topics are visionary. Subsequent work in the late 1990s with colloidal gold particle electrochemistry^6,7^ was perhaps the foundation of what is now known as impact electrochemistry.^8^ Soft impact electrochemistry was then created, although not named, with experiments on single vesicles following separation^9,10^ and then without separation sometime later,^11,12^ and named vesicle impact electrochemical cytometry or intracellular vesicle impact electrochemical cytometry when done inside cells.^13^ In the mix at these times were new applications with immiscible liquid droplets^14^ and repurposing of nanopipettes and nanopores as described below.

Next-generation nanoelectrochemistry has been described by Unwin as having an emphasis on “big data”; its analysis, storage, and curation.^15^ In that paper, which concerned high-throughput analysis and parallelization of nanoelectrochemistry, “intelligent” instruments and experiments; active control of nanoscale systems; and the integration of nanoelectrochemistry and nano- and microscale spectroscopy were discussed. The present meeting was an exemplary overview of these areas.

The field has been traditionally dominated by techniques like scanning electrochemical microscopy (SECM) and scanning electrochemical cell microscopy (SECCM), but the discussion was considerably more diverse in Nanjing. At the 2022 meeting, there were three talks and four posters on SECCM and in 2024 this was similar, with four talks and three posters. However, there were considerably more presentations on nanopore/nanopipette technologies, with six talks and six posters, and two of each on nanoelectrodes.

I will briefly summarize the following areas of nanoelectrochemistry presented at the meeting: nanowires, nanonets, and nanoarrays; nanopores; nanopipettes; spectroelectrochemistry, scanning ion-conductance microscopy (SICM) and light-active processes at nanointerfaces; SECM and SECCM; and nanosensors. One can obtain an overview here and then refer to the referenced papers for more detail and data. I apologize in advance if I miss anyone with a topic a little away from these themes. I will end with a summary of some future (and already present) applications we might think about in this area.

## Nanowires, nanonets, and nanoarrays

Nanoscale materials, including nanoparticles, nanowires, nanotubes, and thin films, have vastly different physical and chemical properties compared to their bulk counterparts. This includes enhanced conductivity, increased surface area, and the potential for more efficient electron transfer, which can make electrochemical reactions faster, more sensitive, and more selective. Several presentations examined or applied these properties.

Transport across membranes is a fundamental process in bioscience. Xia and colleagues presented the development of adjustable graphene oxide (GO) biomimetic membranes to control ion transport (https://doi.org/10.1039/D4FD00149D). In this work, they changed the transport along the axis of the sheet, not across it, by attachment of specific DNA strands to the graphene. Thus, they are examined longitudinal migration. They used multibranched DNA nanowires that were generated *via* the hybridization chain reaction and changed the concentration of the DNA linker strands to variably integrate them on the GO membrane. This allowed them to alter the composition of the membrane and thus change the ion transport along the longitudinal plane of the graphene sheet.

In many areas of biology, it is important to understand the mechanisms and rates of protein denaturation. This provides the structure–function relationships of proteins as well as diseases associated with protein misfolding and aggregation. Zhai and coworkers developed a silicon nitride nanopore and nanonet to then sieve the protein ovalbumin, trapping molecules with electroosmotic flow (https://doi.org/10.1039/D4FD00117F). The processes of denaturation *via* exposure to PbCl_2_ and renaturation of a single ovalbumin molecule were monitored *via* ionic current measurements through these nanonets. They solved the problem of non-specific adsorption of proteins to the SiN_*x*_ nanonets by using polyethylene glycol functionalization. This was used to study the structural changes of single ovalbumin protein molecules while being treated with two molecules that cause them to be denaturatured, Gdn-HCl and Pb^2+^. Thus, it is possible with the nanonets to study denaturation at the single-molecule level.

Mount and coworkers showed a precisely constructed microscale nanoband electrode array for use in flowing systems (https://doi.org/10.1039/D4FD00125G). These arrays are precisely aligned instead of being ensembles and display the advantages of nanoelectrodes with enhanced mass transport and lower double-layer charging.^16–18^ They carried out experimental work and simulations to demonstrate that the microscale nanoband edge array of electrodes has controlled geometry enabling quantitative analysis across a wide range of flow conditions, ranging from passive or evaporative flow to forced advection systems. One application is nanoscale liquid chromatography.

## Nanopores

Nanopore structures are ubiquitous in living systems and have been studied for a variety of analysis approaches over decades. The advent of nanoscale fabrication has completely revolutionized this area and nowhere is it more present than in electrochemical analysis. Nanopore structures provide a means to carry out reactions in limited volumes, to measure with nanoscale resolution, and to search for new reaction chemistries where diffusion is less limiting.

At the meeting, there were 7 posters and 4 presentations that dealt directly with nanopore electrochemistry. Several others used them more indirectly. The emphasis on being able to manage diffusion and trapping at the nanoscale is critical to these experiments.

Yi-Lun Ying presented data showing construction of a reactive K238H-AeL nanopore with histidine sites to carry out single-molecule reactions (https://doi.org/10.1039/D4FD00133H). Each pore had seven histidine residues placed at the 238 site through site-directed mutation. By tuning the physical and chemical properties of the interfaces of the nanochannel walls, mass transport within these artificial 2D channels can be effectively managed. They tuned the physical and chemical properties of the nanopore channel to manage Au(iii) transport and to localize it in the nanopore. They were subsequently able to monitor coordination of the gold to histidine at the single-molecule level and in real time. It is possible these could be placed into nanometer vesicles as reactors.

Nanopores have been used extensively in DNA sequencing.^19,20^ Whereas nanopores have been excellent in sequencing DNA, protein analysis has been challenging. Liang Wang and coworkers have designed a DNAzyme nanopore for electroanalytical measurements of proteins (https://doi.org/10.1039/D4FD00146J). This work uses a molecular sandwich catalytic reaction, and the target is the HIV p24 antigen protein. The DNAzyme-substrate sequences are functional oligonucleotides. The DNAzyme reacts in the nanopore and degrades the nucleic acid substrates to provide enzymatic cleavage products that serve as reporters for reaction. The authors argue that clustered regularly interspaced short palindromic repeats (CRISPR) used for cooperative nanopore detection methods could be made more effective. This might be used in areas such as single-nucleotide polymorphisms and lowering detection specificity in analytical diagnostic methods.

Actis and coworkers showed that a nanopore filled with a polyethylene glycol (PEG) polymer electrolyte enhances detection of nanoparticles at low ionic strength (https://doi.org/10.1039/D4FD00143E). The PEG-based polymer electrolyte only needs to be present inside the nanopore and they shows a significant enhancement in sensitivity for a conical glass nanopore, and specifically for bath-to-nanopore translocating events, when the nanopore electrolyte is composed of 25% (w/v) 35K-PEG in 20 mM KCl. A numerical model was presented showing that the electrical response of the nanopore is sensitive to the position of the polymer electrolyte interface. The goal is to couple the PEG nanopore sensor with nano-impact electrochemistry to create a multimodal analysis and obtain complementary electrochemical reactivity data.

Nanopores have been considered to develop sensors, as templates of biomimetic channels, and as model systems of transport at the nanoscale. These nanopores can restrict ion movement based on charge, and little is known about this effect quantitatively along the conical nano-tip pore. Charges on the pore walls can induce ion selectivity and enhance ionic conductance when compared to uncharged pores. Ziwy and coworkers used three-dimensional modeling to examine the mechanism of applied voltage and pore length in controlling ion selectivity and conductance at single nanopores (https://doi.org/10.1039/D4FD00148F). They also include small nanopore arrays. The work has many conclusions, but perhaps the most important is that ionic transport through nanopores with charged walls is strongly affected by the concentration polarization not only at the pore entrance but also in the pore.

## Nanopipettes

Nanopipettes are not always so different from nanopores, but I make the distinction where the pipette is modified to alter the charge and transit of specific species. This is not necessarily the best means to discriminate, so these two sections are back-to-back. In some nanopipette experiments, the glass surface is chemically modified to create oscillations. There were two presentations at the meeting on nanopipettes and several posters. Nanopipettes hold all the small volume and confinement advantages of pores but are generally constructed of glass or silica.

Xiong and Yu presented work showing a spontaneous current oscillation in nanopipettes with a polyimidazole brush (PvimB) inner coating when a pH gradient is established from inside to outside (https://doi.org/10.1039/D4FD00135D). In this work, a strong negative bias voltage led to spontaneous and periodic current switching and periodic oscillations. They were able to manage the frequency and amplitude of the oscillations by altering the pH gradient and it was also voltage dependent. A long-term goal seems to be to develop iontronic electrical devices and to model transmitter interactions at these interfaces.

Micro and nanopipettes can also be used for substrate delivery. This has been used to deliver lipids, for example, to the insides of cells,^21^ and more classically for delivery of plasmids or pushing the limit smaller to delivery of single molecules to individual cells.^22^ McKelvey and coworkers used a dual-barrel nanopipette to carry out scanning bubble microscopy.^23^ In this method, one barrel is used to create a self-replenishing gas bubble from the reactant and the other is used to carry out scanning ion microscopy. So, here they are using one barrel for nano-delivery. They have used this approach to deliver carbon dioxide to gold electrodes, thereby mapping carbon dioxide production and hydrogen evolution at these electrodes (https://doi.org/10.1039/D4FD00124A). This is aimed at developing a better understanding of the low-temperature electrosynthesis of liquid fuels and chemical feedstocks aimed at a low-carbon economy.

## Spectroelectrochemistry, SICM and light-active processes at nanointerfaces

For this summary, I included spectroscopic, imaging and light-active processes in one category. I will summarize six presentations. Spectroelectrochemistry is a well-established technique. Bringing it to the nanoscale, however, is relatively new, as is imaging. Driving light-based processes with electrochemistry is very new and exciting.

Hiramoto and coworkers presented work with a 1,2-dioleoyl-*sn*-glycero-3-phosphocholine (DOPC) model membrane formed on a planar indium tin oxide (ITO) electrode to carry out electrogenerated chemiluminescence imaging with submicron spatial resolution (https://doi.org/10.1039/D4FD00137K). The electrochemiluminescence (ECL) reaction used was tris(2,2′-bipyridine) ruthenium(ii) with tri-*n*-propylamine as a coreactant. The goal was to examine heterogeneous changes to the membrane following exposure to melittin, an antimicrobial peptide that forms pores in membranes.

Takahashi and coworkers (https://doi.org/10.1039/D4FD00116H) used SICM to visualize the selective toxicity of plasma-activated Ringer's lactate solutions (PALs) toward cancer cells and to determine how they induce apoptosis as reported in 2016.^24^ They focused on SICM time-lapse imaging to follow the timing of normal- and cancer-cell structural changes. These included decreased lamellipodia movement, increased cell volume, and formation of protrusions of several microns on the cell surface. Their data support that PAL exhibits toxicity toward cancer cells and potentially toward normal cells. A novel finding was the limited lamellipodia movement observed in the MCF-7 and MCF-10A cells. The SICM method is a non-contact nanoscale tool for topographic imaging and its value was clear here.

Lu and Zhao used two orthogonal methods, electrochemical current for the mediator and single-molecule fluorescence for the catalytic reduction, to study the EC catalytic reaction of the fluorescent dye, ATTO 647N, in the presence of the mediator phenazine methosulfate (https://doi.org/10.1039/D4FD00126E). The former is translated to a nonfluorescent species on reduction and at low concentrations the on/off switching of the molecular species can be monitored microscopically at submicron and single-molecule resolution.

Gooding and coworkers presented work showing that electrochemistry can be used to drive and modulate single-molecule fluorescence (https://doi.org/10.1039/D4FD00111G). It has been reported that emission from fluorophores can be modulated electrochemically.^23–25^ In the work presented here, the electrochemistry of ferro/ferricyanide was shown to modulate the fluorescence of organic dyes. The oxazine dye ATTO 655 was shown to exhibit fluorescence modulation when the electrochemical potential was switched between −0.8 V and 0.4 V, while another dye, Alexa 488, showed moderate fluorescence intensity change, and another, Alexa 647, exhibited a smaller fluorescence change. Interestingly, these changes are also sensitive to the applied potential and the excitation laser intensity.

## SECM and SECCM

Scanning electrochemical microscopy and more recently scanning electrochemical cell microscopy have been mainstay imaging approaches related to electrochemical processes and nanoelectrochemistry of small structures. The techniques are based in microelectrode technologies created in the 1970s and pushed in the 1980s and 90s, so a lot is known. However, the development of nanoscale materials, the drive to look smaller into the cell, and many other opportunities make the playground for these techniques ever expanding.

At the meeting, there were four talks directly on SECM and SECCM and more referring to them. Hill and Osoro presented work using SECCM to study the nucleation process for silver on carbon and ITO (https://doi.org/10.1039/D4FD00131A). Nucleation and particle growth are fundamental to a lot of electrochemistry and new materials. In the work presented, they showed that surface energies and kinetic rate constants can be measured through the analysis of single-particle nucleation and growth data with SECCM.

Jiang and coworkers presented a new technique based on SECCM called local electrochemical impedance spectroscopy or LEIS (https://doi.org/10.1039/D4FD00122B). This approach allows simultaneous imaging of surface morphology and impedance information with 180 nm resolution. An important aspect of this work was that the integrated SECCM system was established with a preamplifier, in contrast to the traditional lock-in amplifier. This allowed them to recognize smaller picoampere-level currents, opening new doors in sensitivity.

Gaudin and Bentley presented work on measuring the diversity of nanoparticles (https://doi.org/10.1039/D4FD00115J). Our ability to construct and measure nanoparticles is constantly advancing. Here, they discuss that the variability in SECCM responses with nanoparticles is, at least in part, due to full *versus* partial encapsulation of each nanoparticle. Using SECCM to monitor the borohydride oxidation reaction at gold nanoparticles on highly ordered graphite, they show particles displaying a fluctuating current response. These might be the particle moving up and down on the electrode surface or repeatedly making and breaking electrical contact. It is also possible that particles are carried by the SECCM tip and this would also lead to a fluctuating current. The paper is presented much as a discussion and the authors argue for not excluding anomalies in single impact data analysis with the point that these so-called anomalies are likely real impact phenomena.

Ren and coworkers used SECCM to study the effect of the local electrolyte environment in electrocatalytic reactions (https://doi.org/10.1039/D4FD00080C). They carry out experiments to measure the transport level owing to electroosmotic flow and electromigration in the nanopipette used for SECCM. Here they used electrochemistry and fluorescence imaging of dual-barrel nanopipettes in SECCM and combined this with finite element simulations to visualize and quantify the processes. They showed that at neutral pH, the main contribution was electromigration for highly charged species and both electromigration and electroosmotic flow for singly charged species. They also suggest that these measurements might be used to develop strategies for precise modulation of species flux at the nanoscale.

## Nanosensors

An area of application of nanotechnology comprises making measurements in small regions of biological systems. One such area of high heterogeneity at the micrometer and nanometer level is the brain. There have been many sensors for non-electroactive neurotransmitters, and glutamate is often the target of this research; however, these have rarely approached the nanometer scale, usually for reasons of sensitivity. Shen and coworkers developed an enzyme-based nanosensor for glutamate that shows promise here (https://doi.org/10.1039/D4FD00138A). They used a 210 nm radius planar Pt nanoelectrode with glutamate oxide deposition for glutamate detection.

Caniglia and coworkers have developed a novel SECM probe with platinum black deposition on recessed gold disk electrodes with diameters of 1 μm, 500 nm, and 250 nm (https://doi.org/10.1039/D4FD00136B). The sensor can be used to monitor hydrogen evolution and is aimed at light-driven water splitting reactions in energy devices. Deposition leads to an increased electrochemically active surface area owing to the hemispherical geometry. These probes allow effective quantification of H_2_ with concentrations up to 300 mM and were tested on two different model systems for photocatalytic and electrocatalytic H_2_ evolution.

Fu and coworkers have developed a Nafion-coated nanoporous gold electrode as an electrochemical aptamer-based biosensor (https://doi.org/10.1039/D4FD00144C). Nanoporous gold electrodes were designed with 10–30 nm pores to obtain a randomly distributed nanoporous structure. These pores are too small for Nafion to enter. Thus, the target molecule is allowed to pass through the membrane and interact with the aptamers immobilized on the inner pores, while the Nafion stays as a barrier to anions. The authors here developed a sensor for doxorubicin, a chemotherapy drug.

## Perspectives on the future of nanoelectrochemistry

Nanoelectrochemistry has grown from a novelty in the 1980s to becoming a broad and important topic. Fundamental work is needed in many areas as we move smaller and more complicated. In addition, many techniques, when developed without a specific application goal, take the path of least resistance. There is a need, however, to apply this wonderful technology to important topics. Potential applications, some of which were touched on at the meeting, include molecular delivery, metabolite labeling in nano-environments, membrane oscillation monitoring, and sensors for single cells and organelles.

The recent discovery of a new organelle^26^ and the electrochemical formation of hydrogen peroxide at droplets and protein aggregates^27–29^ are exciting areas where nanoelectrochemistry will play an important role. When coupled with the discovery of reactive oxygen formed by protein-aggregate organelles like stress granules^30^ and their effect on nano vesicles,^31^ a new frontier opens for nanoelectrochemical measurements.

To truly reach the promise of new fundamental understanding at the nano-domain of physical and biological materials, multimodal approaches will be required. Surface enhanced Raman and tip-enhanced Raman spectroscopies, SERS and TERS, respectively, are important tools that are progressing now to become extremely important at the nano-domain. The orthogonal information from light spectroscopy and electrochemistry is extremely powerful, and as shown in the presentation by Gooding and coworkers, the fundamental underlying principles of both can be related. Other multimodal methods should be considered, like the combination with mass spectrometry where desorption electrospray ionization (DESI) can be carried out to create ions at atmospheric pressure and they can then be analyzed. Although DESI and even nano-DESI^32^ are not yet available at the nano-domain for spatial resolution, they can be useful tools when used in combination with electrochemistry for mechanistic studies.

The use of computer modeling and artificial intelligence (AI) will obviously become important in areas from experimental design to data interpretation and modeling. In fact, at the meeting, Zhao and coworkers showed the use of a convolutional neural network to develop an algorithm for step detection in single-entity measurements (https://doi.org/10.1039/D4FD00130C), and Lin *et al.* have developed AI-assisted imaging fusion methods to improve the contrast and resolution of SECM images of tissues.^33^

I used AI (ChatGPT) to provide a list of Challenges and Opportunities in Nanoelectrochemistry and then a list of Future Directions to complement my thoughts.

Challenges and opportunities of nano electrochemistry listed by ChatGPT:

• Scalability and cost: while nanomaterials show great promise, the manufacturing of nanoscale devices on a large scale is still challenging. The cost and complexity of producing high-quality, reproducible nanomaterials and integrating them into practical devices remain significant barriers.

• Stability and durability: at the nanoscale, materials can be more susceptible to degradation over time due to their high surface energy and increased reactivity. Ensuring the long-term stability and durability of nanoelectrochemical systems is critical for their practical use in energy storage, sensing, and other applications.

• Understanding fundamental mechanisms: the complex interplay between size, surface structure, and electrochemical behavior at the nanoscale is not yet fully understood. More fundamental research is needed to uncover the detailed mechanisms governing charge transfer, electrocatalysis, and other electrochemical processes at the nanoscale.

• Environmental and health concerns: as with any new technology, there are concerns regarding the potential environmental and health impacts of nanoscale materials. The toxicity of certain nanoparticles and their behavior in the environment must be carefully considered to ensure safe implementation of nanoelectrochemical technologies.

Future directions according to ChatGPT:

• Multifunctional nanomaterials: there is an increasing interest in developing multifunctional nanomaterials that combine several properties, such as high conductivity, catalytic activity, and biocompatibility. For example, hybrid nanomaterials that combine metals, polymers, and semiconductors could be used in advanced electrochemical applications, including artificial photosynthesis or energy harvesting.

• Advanced fabrication techniques: as the need for precision in the design of nanoelectrochemical systems grows, so does the need for advanced fabrication techniques, such as bottom-up assembly, self-assembly, and 3D printing of nanostructures. These methods could allow for better control over material properties and the creation of highly customized devices.

• Integration with other technologies: nanoelectrochemical systems could be integrated with other emerging technologies such as artificial intelligence (AI), the Internet of Things (IoT), and machine learning. For example, AI algorithms could be used to predict the behavior of electrochemical systems, optimizing their performance in real time.

Whereas this list is interesting, it is a bit centric on the AI, and a bit general. There are, however, a great deal of opportunities in this area of research and development.

In conclusion, nanoelectrochemistry represents an exciting field that is evolving rapidly on the decade time-scale. There is great potential for the transformation of the energy, environmental protection, healthcare, and electronics industries with nanoelectrochemistry. The challenges and hurdles to overcome include better scalability, stability, and fundamental understanding, before these technologies can actually be widely adopted. Looking at the progress from these Faraday meetings from 2016 to now, I predict we will see more exciting developments as research continues over perhaps the next five to ten years.

Finally, in reading the introduction by Lane Baker (https://doi.org/10.1039/D4FD00159A), I agree and disagree with discussion relating to the bridges between people. I agree that a meeting like the Faraday Discussion needs extra contact. However, I think we need to accept the future and look for better ways to bridge these gaps online. We need to especially look for these for early career scientists. There are simply too many meetings and too much travel. Time and our climate are precious commodities. The pandemic, which I argue is not really over, has provided a window into the future and we need to take advantage of this. Whether we like it or not, this is the future, and we need to adapt.

## Data availability

There are no new data to share in this article as it is a summary.

## Conflicts of interest

There are no conflicts of interest to declare.

## References

[cit1] Bohn P. (2018). Faraday Discuss..

[cit2] Unwin P. (2022). Faraday Discuss..

[cit3] Fleischmann M. , Pons S., Rolison D. R. and Schmidt P. P., Ultramicroelectrodes, Datatech Systems, Morganton, NC, 1987

[cit4] Wightman R. M. (1981). Anal. Chem..

[cit5] https://ia601901.us.archive.org/15/items/DTIC_ADA328739/DTIC_ADA328739_text.pdf

[cit6] Ung T., Giersig M., Dunstan D., Mulvaney P. (1997). Langmuir.

[cit7] Chen S., Ingram R. S., Hostetler M. J., Pietron J. J., Murray R. W., Schaaff T. G., Khoury J. T., Alvarez M. M., Whetten R. L. (1998). Science.

[cit8] Fan F.-R. F., Bard A. J. (2008). Nano Lett..

[cit9] Omiatek D. M., Santillo M. F., Heien M. L., Ewing A. G. (2009). Anal. Chem..

[cit10] Omiatek D. M., Dong Y., Heien M. L., Ewing A. G. (2010). ACS Chem. Neurosci..

[cit11] Cheng W., Compton R. G. (2014). Angew. Chem., Int. Ed..

[cit12] Dunevall J., Fathali H., Najafinobar N., Lovric J., Wigström J., Cans A.-S., Ewing A. G. (2015). J. Am. Chem. Soc..

[cit13] Li X., Majdi S., Dunevall J., Fathali H., Ewing A. G. (2015). Angew. Chem., Int. Ed..

[cit14] Kim B.-K., Boika A., Kim J., Dick J. E., Bard A. J. (2014). J. Am. Chem. Soc..

[cit15] Xu X., Valavanis D., Ciocci P., Confederat S., Marcuccio F., Lemineur J.-F., Actis P., Kanoufi F., Unwin P. R. (2023). Anal. Chem..

[cit16] Arrigan D. W. M. (2004). Analyst.

[cit17] Cox J. T., Zhang B. (2012). Annu. Rev. Anal. Chem..

[cit18] Murray R. W. (2008). Chem. Rev..

[cit19] Benner S., Chen R. J. A., Wilson N. A., Abu-Shumays R., Hurt N., Lieberman K. R., Deamer D. W., Dunbar W. B., Akeson M. (2007). Nat. Nanotechnol..

[cit20] Manrao E. A., Derrington I. M., Laszlo A. H., Langford K. W., Hopper M. K., Gillgren N., Pavlenok M., Niederweis M., Gundlach J. H. (2012). Nat. Biotechnol..

[cit21] Aref M., Ranjbari E., Romiani A., Ewing A. G. (2020). Chem. Sci..

[cit22] Chau C. C., Maffeo C. M., Aksimentiev A., Radford S. E., Hewitt E. W., Actis P. (2024). Nat. Commun..

[cit23] Monteiro J., McKelvey K. (2024). Anal. Chem..

[cit24] Lou Z., Li P., Han K. (2015). Acc. Chem. Res..

[cit25] Doppagne B., Chong M. C., Bulou H., Boeglin A., Scheurer F., Schull G. (2018). Science.

[cit26] Xu C., Xu J., Tang H. W., Ericsson M., Weng J.-H., DiRusso J., Hu Y., Ma W., Asara J. M., Perrimon N. (2023). Nature.

[cit27] Chamberlayne C. F., Zare R. N. (2022). J. Chem. Phys..

[cit28] Wei Z., Li Y., Cooks R. G., Yan X. (2020). Annu. Rev. Phys. Chem..

[cit29] Dai Y., Chamberlayne C. F., Messina M. S., Chang C. J., Zare R. N., You L., Chilkoti A. (2023). Chem.

[cit30] Hu K., Relton E., Locker N., Phan N. T. N., Ewing A. G. (2021). Angew. Chem..

[cit31] Gu H., Gu C., Locker N., Ewing A. G. (2024). Angew. Chem..

[cit32] Roach P. J., Laskin J., Laskin A. (2010). Analyst.

[cit33] Lin Y.-H., Tsai C.-N., Chen P.-F., Lin Y.-T., Darvishi S., Girault H. H., Lin T.-Y., Liao M.-Y., Lin T.-E. (2022). ACS Meas. Sci. Au.

